# Mutation in Integrin-Linked Kinase (ILK^*R211A*^) and Heat-Shock Protein 70 Comprise a Broadly Cardioprotective Complex

**DOI:** 10.1371/journal.pone.0077331

**Published:** 2013-11-18

**Authors:** Alexandra Traister, Mark Walsh, Shabana Aafaqi, Mingliang Lu, Xiaojing Dai, Mark R. Henkleman, Abdul Momen, Yu-Quing Zhou, Mansoor Husain, Sara Arab, Sara Piran, Gregory Hannigan, John G. Coles

**Affiliations:** 1 Division of Cardiovascular Research, Hospital for Sick Children, Toronto, Canada; 2 Cell Adhesion Signaling Laboratory, Monash Institute of Medical Research, Melbourne, Australia; 3 Mouse Imaging Facility, Toronto Centre for Phenogenomics, Toronto, Canada; 4 Division of Cellular & Molecular Biology, University Health Network, Toronto, Canada; UAE University, Faculty of Medicine & Health Sciences, United Arab Emirates

## Abstract

**Rationale:**

Integrin-linked kinase (ILK) has been proposed as a novel molecular target that has translational potential in diverse cardiac diseases, since its upregulation promotes a broadly cardioprotective phenotype. However, ILK has been implicated as both a cardioprotective and oncogenic target, which imposes therapeutic constraints that are generally relevant to the translational potential of many kinases.

**Objective:**

To study the cardioprotective properties of the activation-resistant, non-oncogenic, mutation of ILK (ILK^*R211A*^) against experimental MI *in* *vivo* and Doxorubicin induced apoptosis *in* *vitro* and it’s relationships to stress induced heat shock proteins.

**Methods/Results:**

The transgenic mouse heart over-expressing a point mutation in the ILK pleckstrin homology (PH) domain (Tg^*R211A*^) exhibits a highly cardioprotective phenotype based on LAD-ligation-induced MI reduction *in* *vivo*, and on protection against doxorubicin (DOX)-induced cardiomyocyte apoptosis when overexpressed in human induced pluripotent stem cell (iPS)-derived cardiomyocytes *in* *vitro*. Intriguingly, the degree of cardioprotection seen with the ILK^*R211A*^ mutation exceeded that with the ILK^*S343D*^ mutation. Microarray and immunoprecipitation analyses revealed upregulation of expression levels and specific binding of ILK^*WT*^, ILK^*S343D*^ and ILK^*R211A*^ to both constitutively active heat-shock protein 70 (Hsc70) and inducible Hsp70 in response to MI, and to acute ILK overexpression in iPSC-cardiomyocytes. ILK-mediated cardioprotection was shown to depend upon Hsp70 ATPase activity.

**Conclusions:**

These findings indicate that wild type ILK and the non-oncogenic ILK^*R211A*^ mutation comprise a cardioprotective module with Hsp/c70. These results advance a novel target discovery theme in which kinase mutations can be safely engineered to enhance cardioprotective effects.

## Introduction

Little progress has been made in the past decade on mitigating the increasingly large clinical and economic impacts of heart failure (HF). Large Phase III trials have so far failed to identify safe and clearly effective therapies based on molecular mechanisms in HF[1]. Integrin-linked kinase (ILK) has been proposed as a novel molecular target that has translational potential in diverse cardiac diseases, since its upregulation promotes a broadly cardioprotective phenotype[2-4]. ILK activation by growth factor stimulation is normally regulated downstream of phosphatidylinositol 3-kinase (PI3K) by phosphatidylinositol (3–5)-trisphosphate (PIP3), which binds with the central pleckstrin homology (PH)-like domain of ILK[5]. ILK^*R211A*^ carries a synthetic arginine (R) to alanine (A) point mutation in its PH domain which makes it deficient in membrane PIP3 binding and renders it resistant to PI3K-dependent activation[2,6], [7]. The ILK^*R211A*^ mutation is of translational interest since wild type ILK, as is the case with other cytoprotective Ser/Thr kinases such as PKB/Akt[8], has been implicated as an oncogene[7,9-11]. To explore the cardioprotective properties of ILK, we generated and performed LAD ligation in two distinct transgenic mouse models: one using a constitutively-active serine (S) to aspartate (D) substitution in the putative autophosphorylation site of the human ILK gene (Tg^*S343D*^)[12]; the other expressing the activation-resistant Tg^*R211A*^ variant in the myocardium[2,6,7] .

Here we show that the presumed activation-resistant, non-oncogenic ILK^*R211A*^ mutation[6,7] is cardioprotective against experimental MI *in vivo* and Doxorubicin-induced apoptosis in human cardiomyocytes *in vitro*. We also show that both wild type and R211A ILK mutations are client molecules of the constitutively active and stress-inducible heat-shock protein 70 (Hsc/p70), and that both ILK and Hsp70 are required for the broadly cardioprotective phenotype induced by ILK upregulation.

## Results

### Transgenic Myocardial Expression of ILK^*R211A*^ is Protective Against MI

**Figure 1:**
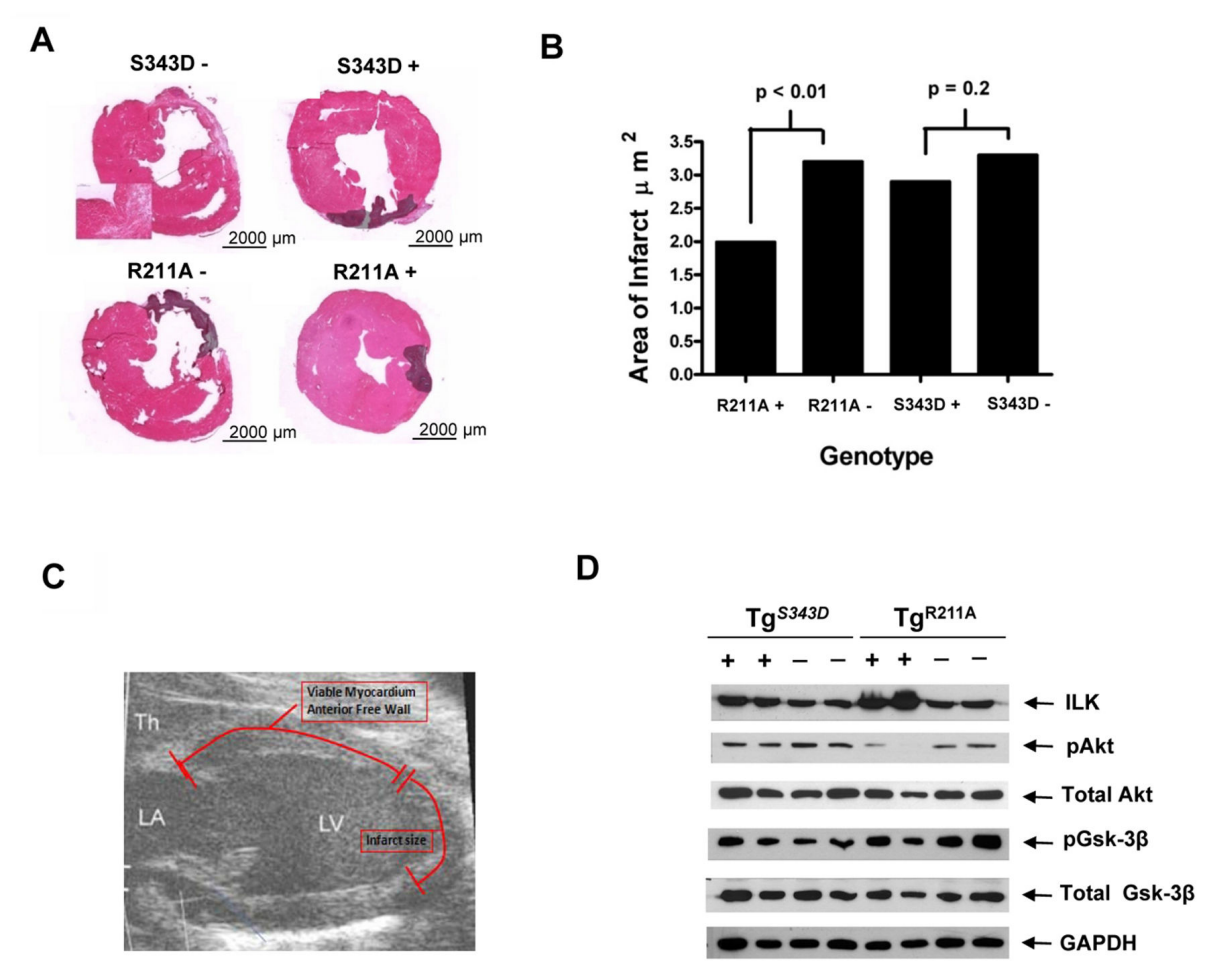
ILK^*R211A*^ Improves Post-Infarct Remodeling. **A**, H & E staining of axial sections 28 days post LAD ligation in transgenic (Tg) mice with cardiac-specific over-expression of constitutively-active (S343D+) or R211A+ mutation and littermate controls (S343D- and R211A-). Scale bar, 200 µm (high magnification), 2000 µm (low magnification). **B**, Planimetric calculations indicate a significant reduction in infarct size in R211A genotype, p(ANOVA) <0.01, and a similar trend in the S343D genotype. **C**, Echocardiographic measurements show a reduction in infarct size and reciprocal increase in region of viable myocardium in favor of R211A (p=0.04; p=0.001, respectively) and S343D (p=0.12; p=0.05) genotypes as compared to their littermate controls. Detailed measurements are provided in Tables S1 and S2, Online Supplement. **D**, Western blot showing the protein levels of phosphorylated Akt (pAkt) and Gsk-3β and total amounts of Akt and Gsk-3β in post-MI border zone myocardial lysates derived from two Tg^*S343D*^ and two Tg^*R211A*^ mice (+) and their littermate controls (-). GAPDH was used as a loading control.

The cardiac phenotypic responses to LAD ligation were determined in transgenic mice conveying the ILK^*R211A*^ or ILK^*S343D*^ mutations as assessed at 28 days to allow post-MI remodeling to occur. The ILK^*R211A*^ mouse exhibited a cardioprotective phenotype against LAD ligation that was greater than that in the activated ILK^*S343D*^ genotype (Figure 1A, B and Tables S1, S2). Based upon echocardiographic measurements of akinetic LV wall segment length, Tg^*R211A*^ mice sustained significantly smaller infarcts compared to littermate controls and to that in Tg^*S343D*^ mice [p(ANOVA) <0.05 for both comparisons] (Figure 1C). Tg^*R211A*^ mice exhibited a significant increase in stroke volume (p=0.04) and decrease in heart rate (p=0.02) relative to that of the Tg^*S343D*^ genotype at 28 days post MI consistent with improved cardiac function (Table S1). Thus, Tg^*R211A*^ mice exhibited significantly smaller infarcts and improved function in response to LAD ligation relative to both littermate controls and to Tg^*S343D*^ mice.

Since ILK is a protein Ser/Thr kinase that causes phosphorylation of Akt/PKB on Ser473 and glycogen synthase kinase-3β (Gsk-3β) on Ser9[11], and the ILK^*R211A*^ mutation is thought to be either null or inhibitory to canonical ILK signaling[6,7,13], we measured the phosphorylation status of these ILK prosurvival targets in border zone of post-MI myocardium. Interestingly, ILK^*R211A*^ lysates showed a clear downregulation in the p(ser473)-Akt signal (Figure 1D). The findings that the activation-resistant ILK^*R211A*^ mutation was more potently cardioprotective than that of the catalytically-active ILK^*S343D*^, yet is suppressive to Akt activation, suggest that improved post MI remodeling is due to the scaffolding[14,15] rather than catalytic properties[5,6] resulting from ILK^R211A^ upregulation.

### Wild Type and ILK Mutations Form Heat-Shock Protein 70 Complexes

**Figure 2:**
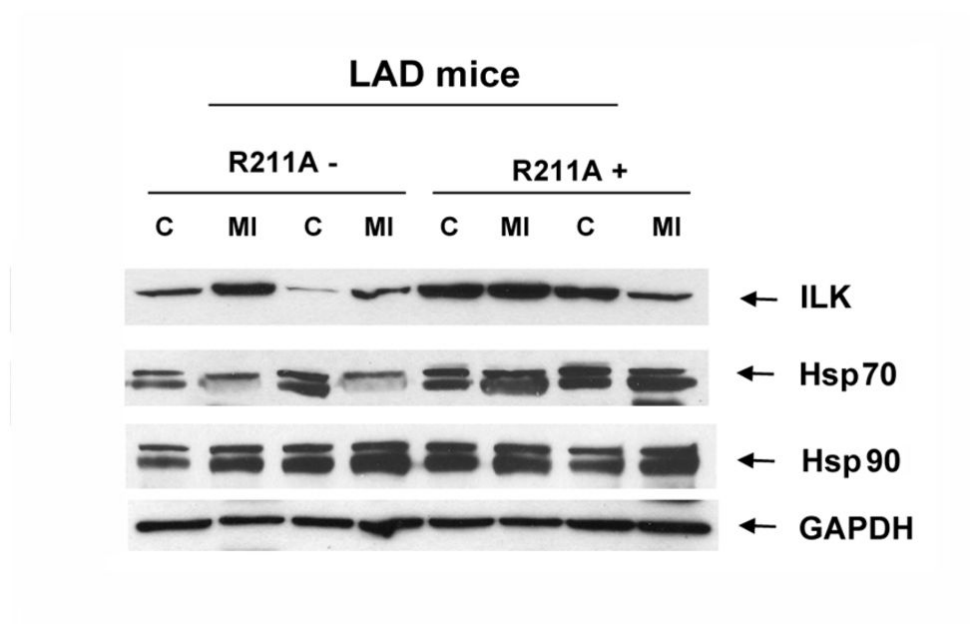
ILK^*R211A*^ induces Hsp70 expression in the infarct zone of LAD-ligated mice. Western blot analysis of the protein levels of ILK, Hsp70 and Hsp90 in the lysates of myocardial infarct zone (MI) and control, viable, non infarcted myocardial zone (C) obtained from the hearts of two 28 days post LAD ligated Tg^*R211A*^ mice (+) and their littermate controls (-). GAPDH served as a loading control.

To explore the mechanism through which the ILK^*R211A*^ mutation in transgenic mice hearts conferred a greater degree of cardioprotection, microarray analysis was performed in the hearts of Tg^*R211A*^ and Tg^*S343D*^. ILK^*R211A*^ mutation compared to littermate controls elicited a robust heat shock mRNA response (Figures S1 and S2; Table S3), featuring dominant activation of heat shock protein 70 (Hsp70) (~3-fold) that was most pronounced in the ILK^*R211A*^ as compared to the ILK^*S343D*^ mutation (not shown). The top Gene Ontology (GO) Biological Processes categories significantly upregulated in the R211A genotype included protein folding, response to stress, chaperone cofactor-dependent protein folding, endoplasmic reticulum stress, and post-translational protein folding (p < 0.001 for all GO categories). In line with the gene expression data, the protein expression levels of Hsp70 measured in the infarct zone of LAD-ligated Tg^*R211A*^ hearts were higher than that in littermate controls (Figure 2).

**Figure 3:**
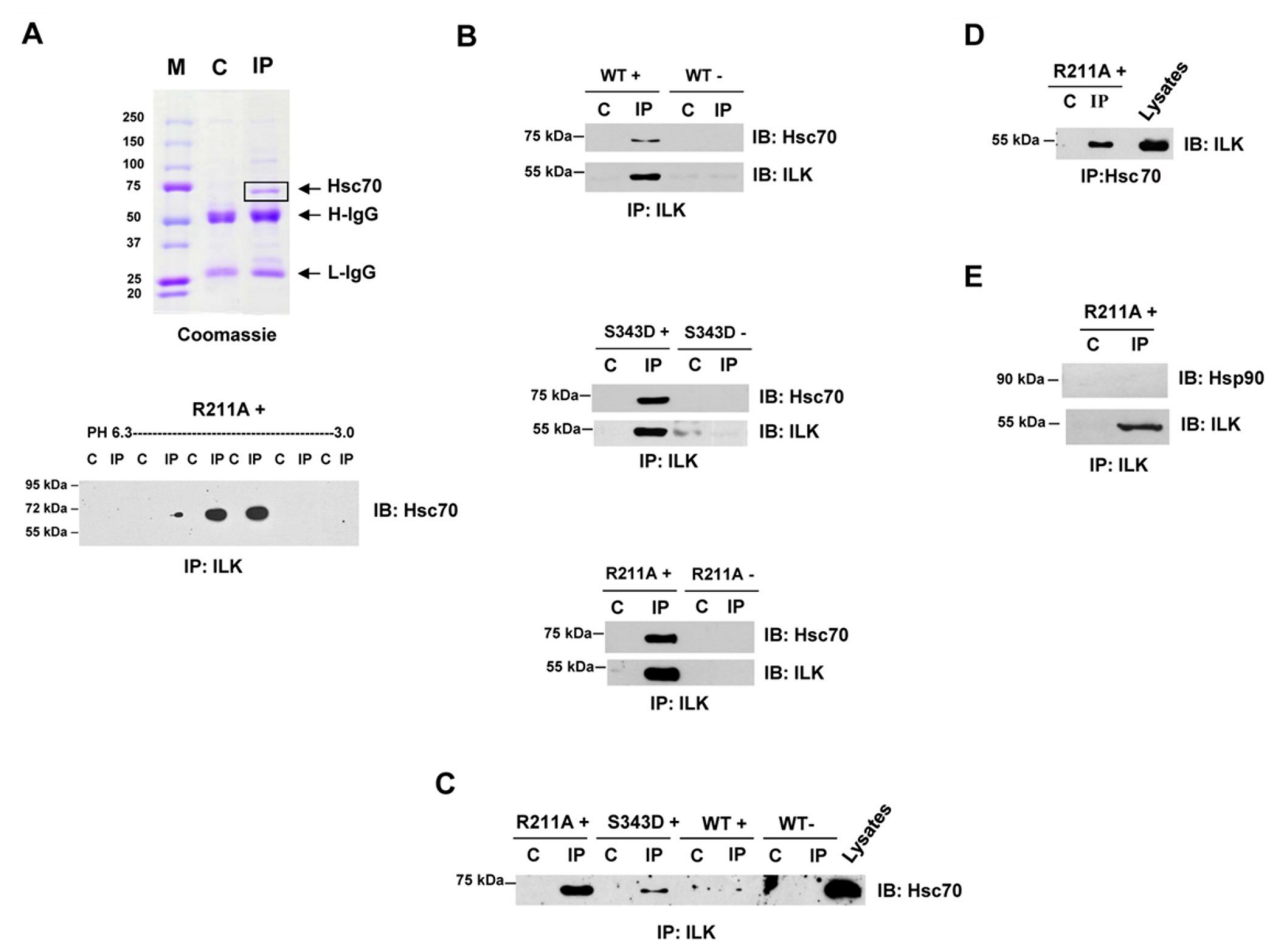
ILK forms a complex with Hsc70. **A**, (upper panel) SDS gel of ILK immunoprecipitates (IP) stained with Coomassie brilliant blue identified a major band at the size of 70 kD, which was confirmed by mass spectrometry as the mouse orthologue of heat-shock cognate protein 70 (Hsc70, also known as Hsp73). IP of ILK^*R211A*^ binds specifically to Hsc70 in 2D Isoelectric Focused (IEF)/SDS gel (lower panel). Myocardial lysates obtained from Tg^*R211A*^ hearts were immunoprecipitated using anti ILK rabbit Ab (IP) and control rabbit IgG (C) and then loaded into wells of an (IEF; pH 3.0-6.3) gel. ILK IP and C lanes were cut and inserted into wells of SDS gel, transferred and stained in Western blot using anti-Hsc70 goat Ab. **B**, ILK IPs derived from myocardial lysates of transgenic mice with cardiac-specific over-expression of ILK wild type (WT+), constitutively-active (S343D+) or activation resistant R211A+ mutations and their littermate controls (WT-, S343D- and R211A-) were probed with anti-Hsc70 antibodies. **C**, Western blot analysis demonstrating ILK IPs derived from myocardial lysates of transgenic mice showing gradient binding of ILK with Hsc70 which was the highest in R211A+, intermediate in S343D+, and lowest in WT+. **D**, Reciprocal analysis showing ILK binding in Hsc70 IP. **E**, ILK does not Co-IP with Hsp90. Co-IPs was performed in triplicate on independent samples and representative blots are shown.

1D and 2D SDS gels of ILK immunoprecipitates in ILK^*R211A*^ lysates were performed to identify ILK-interacting proteins (Figure 3A). The human orthologue of the major band revealed by Coomassie blue staining was recognized using mass spectrometry as heat-shock cognate protein 70 (Hsc70, also known as Hsp73). The specificity of wild type ILK, ILK^*S343D*^, and that of ILK^*R211A*^ binding to both Hsc70 and Hsp70 was demonstrated by co-immunoprecipitation (co-IP) with anti ILK antibody and confirmed by reciprocal co-IP using anti Hsp/c70 antibodies (Figures 3B). Among the ILK variants, ILK^*R211A*^ showed the highest levels of co-IP with Hsc/p70 (Figure 3B, D). We also analysed if other heat shock proteins such as Hsp90 interact with ILK. To this end we performed co-IP using anti Hsp90 antibodies in the lysates of Tg^*R211A*^ hearts, in which the strongest binding of ILK with Hsp/c70 was observed. As compared to Hsp/c70, no binding to Hsp90 was identified (Figure 3E). These results suggest that both ILK^*S343D*^ and ILK^*R211A*^ are specific client molecules of Hsc/p70.

### Cytoprotective Properties of ILK Depend Upon an ILK-Hsc/p70 Complex

**Figure 4:**
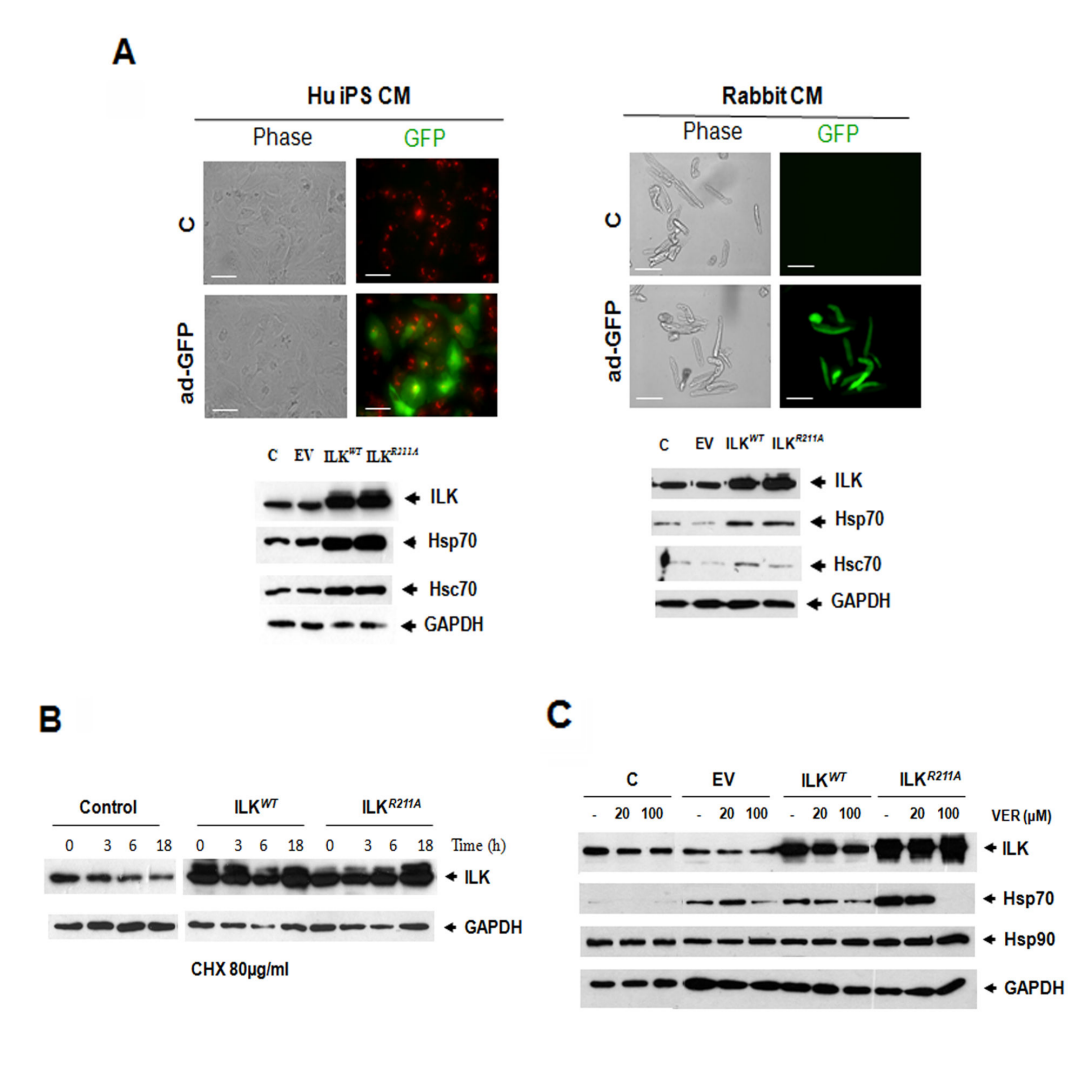
ILK Induces Hsc70 and Hsp70 Expression in Human Cardiomyocytes. **A**, (top panels) Phase contrast and fluorescent microscopy images of human IPSC-derived cardiomyocytes (Hu iPS-CMs) (on the left) and primary rabbit cardiomyocytes (on the right) transduced with GFP-linked vector (ad-GFP) versus non transduced cells (C). The Hu iPS-CM cells express monomeric red fluorescent protein and blasticidin resistance under the control of the α-myosin heavy chain promoter that allows simultaneous cardiomyocyte purification and identification. Green fluorescence indicates efficient transduction of ILK constructs. Scale bar, 80 µm. (bottom panels) Western blots showing expression levels of ILK, Hsp70 and Hsc70 in Hu iPS-CMs (on the left) and in primary rabbit cardiomyocytes (on the right) transduced with ILK^*WT*^ and ILK^*R211A*^ as compared to non-transduced cells and cells transduced with the vector bearing GFP only. GAPDH was used as a loading control. **B**, Immunoblot for ILK expression levels in Hu IPS-CMs transduced with ad-ILK^*WT*^ and ad-ILK^*R211A*^ as compared to non-transduced cells following treatment with cycloheximide (CHX) at 80 µM for 0, 3, 6 and 18 hours. **C**, Western blot analysis showing the protein levels of ILK, Hsp70 and Hsp-90 in Hu iPS-CMs transduced with ad-GFP, ad-ILK^*WT*^, ad-ILK^*R211A*^ and in non-transduced cells (C) following treatment with indicated doses of Hsc/p70-ATPase inhibitor (Ver-155008) for 24 hours. GAPDH was used as a loading control. Each experiment was performed at least three times on independent samples and one representative blot is shown.

The effect of overexpression of the wild type ILK (ILK^*WT*^) and ILK^*R211A*^ mutant was also evaluated in human induced pluripotent stem cell (iPS)-derived human cardiomyocytes[16,17] *in vitro*. Cardiomyocytes generated using this procedure are highly purified (>95%) and exhibit the structural, contractile and electrophysiological properties of bona fide cardiomyocytes[17]. Adenoviral overexpression of ILK^*R211A*^ and ILK^*WT*^ resulted in an increased expression of ILK and that of Hsc70 and Hsp70 protein levels (hereafter referred to as Hsp70 since their expression levels were found to be similar under all experimental conditions) (Figure 4A). The induction of Hsc/p70 in response to ILK^*R211A*^ and ILK^*WT*^ was also observed in rabbit cardiomyocytes (Figure 4A, right panel) thus ruling out Hsp70 induction secondary to cellular reprogramming effects. Furthermore, in both, human iPSC-derived cardiomyocytes and rabbit cardiomyocytes, ILK expression levels were significantly higher following ILK^*R211A*^ transduction as compared to those in ILK^*WT*^ overexpressing cells (Figures 4A). We also analysed if Hsp70 upregulation in ILK overexpressing cells was associated with an increase in ILK stability. Addition of the protein translational inhibitor cycloheximide showed that the rate of degradation of adenovirally-overexpressed ILK (ILK^*WT*^ and ILK^*R211A*^) was reduced compared to that of endogenous ILK, indicating an increased chaperone-mediated proteolytic stability in ILK overexpressing cells (Figure 4B). The addition of the novel adenosine-derived inhibitor of Hsc/p70-ATPase activity (Ver-155008)[18] caused a dose-dependent reduction in Hsp70 expression that was most evident in the ILK^*R211A*^ expressing cells (Figure 4C), suggesting that the induction of Hsc/p70 by ILK (WT and R211A) depends upon a functional Hsp70-ATPase chaperone cycle. 

### ILK^*WT*^ and ILK^*R211A*^ Protect Against DOX-induced Cardiotoxicity

**Figure 5:**
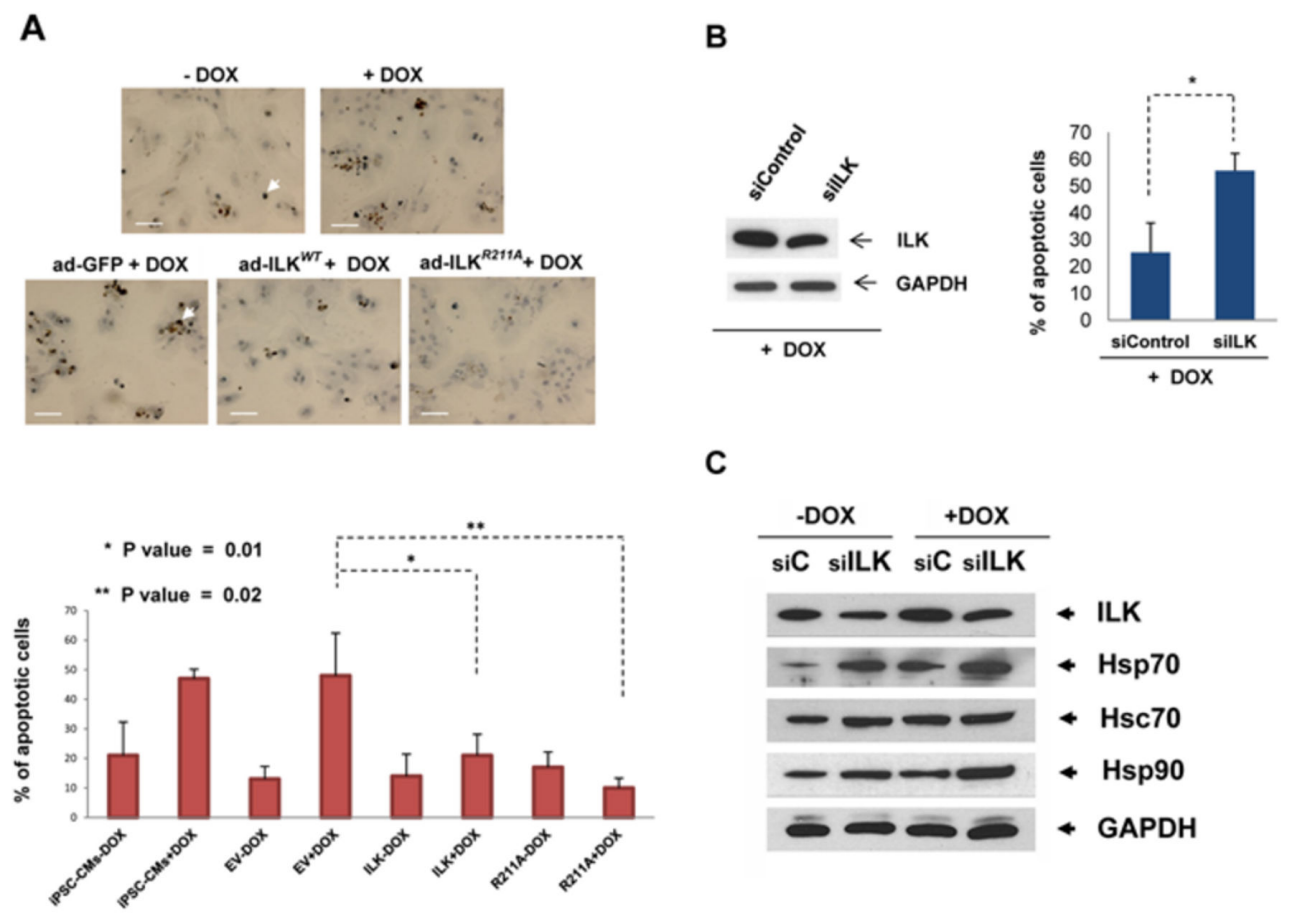
ILK protects against DOX-induced apoptosis. **A**, (top panel) Immunohistochemical staining for TUNEL-positive apoptotic nuclei in Hu iPSC-CMs transduced with ad-ILK*WT*, ad-ILK*R211A*, ad-GFP and in non-transduced cells following exposure to 1µM DOX for 24 hours. Scale bar, 100 µm. Arrows point to TUNEL-positive apoptotic nuclei. (bottom panel) Quantitative analysis showing the percentage of TUNEL-positive cell in Hu iPS-CMs after adenoviral infection and exposure to DOX. Bar graphs represent mean values ±SD, n=10 (random fields). * *P* < 0.01, ** *P* <0.02 by student *t* test. **B**, Western blots indicating the expression levels of ILK (on the left) and quantitative analysis showing the percentage of TUNEL-positive cell (on the right) in Hu iPS-CMs treated with ILK siRNA (siILK) or scrambled siRNA (siControl) following exposure to 1µM DOX for 24 hours (+DOX). Bar graphs represent mean values ±SD, n=10 (random fields). * *P* < 0.01 by student *t* test. **C**, Immunoblot showing the expression levels of ILK, Hsp70, Hsc70 and Hsp90 in Hu iPS-CM transduced with ILK siRNA (siILK) or scrambled siRNA (siC) following exposure to 1µM DOX for 24 hours (+DOX) as compared to DOX untreated cells (-DOX).

To further investigate the Hsp/c70 dependent cardioprotective effect of ILK we used Doxorubicin (DOX), an effective and frequently used chemotherapeutic agent for various malignancies, which causes both an acute and late-onset, dose-limiting cardiomyopathy[19,20]. DOX (1μM) was shown to induce ~50% apoptosis rate (EC_50_) in empty vector control-treated iPS-derived cardiomyocytes (Figure 5A). Transduction of ILK^*WT*^ and ILK^*R211A*^, but not vector alone, resulted in a significant reduction in the rate of apoptosis in ILK overexpressing cells following DOX exposure (Figure 5A).

**Figure 6:**
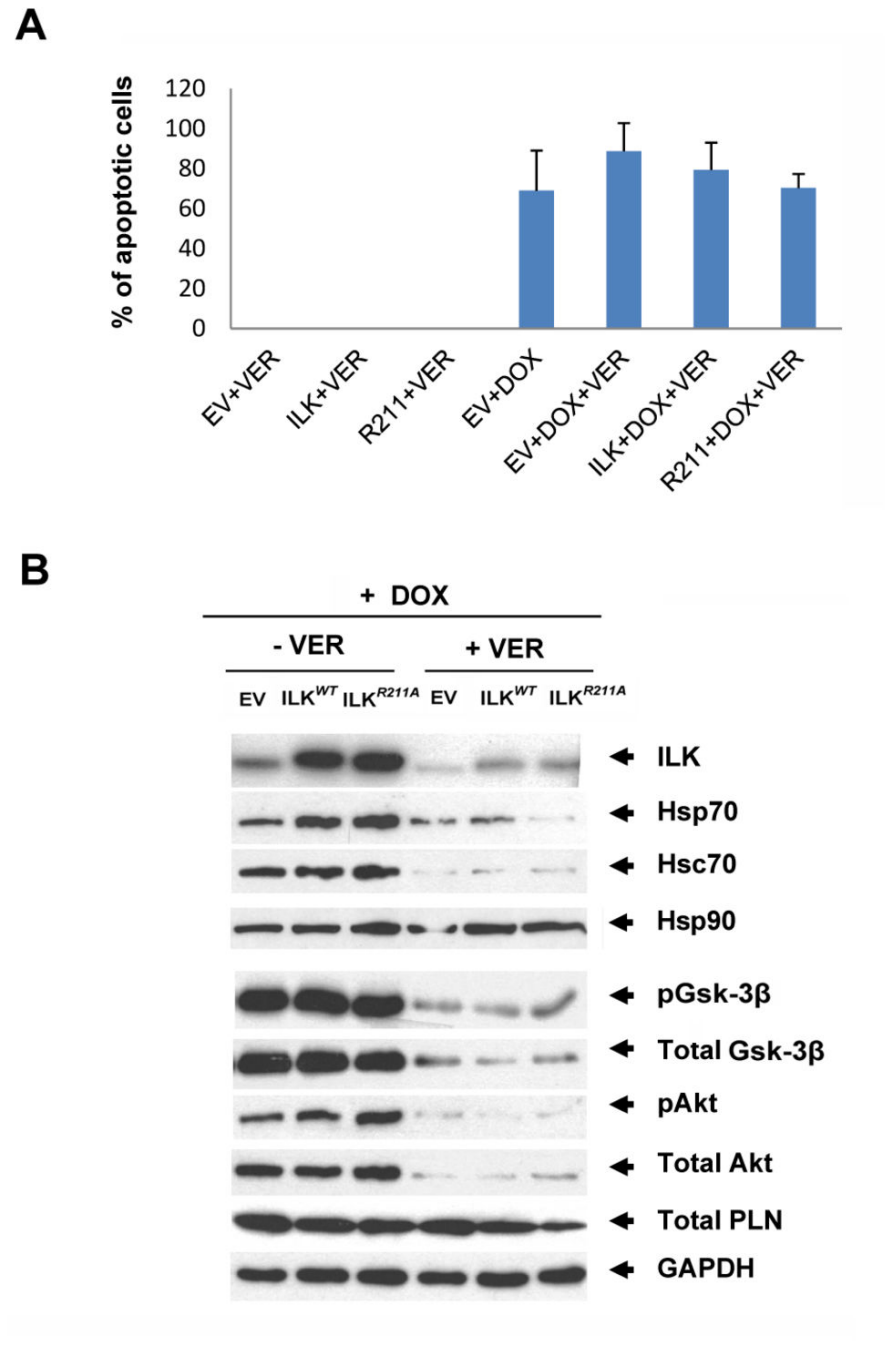
Hsc/p70 activity is required for cardioprotective effect of ILK (WT and R211A) against DOX-induced apoptosis. **A**, Percentage of TUNEL-labelled apoptotic nuclei in Hu iPS-CMs transduced with ad-GFP, ad-ILK^*WT*^ and ad-ILK^*R211A*^ and then treated for 24 hours with 40 µM of Hsc/p70-ATPase inhibitor Ver-155008 in the presence or absence of 1 µM DOX. Ver-155008 was added one hour prior to exposure to DOX. Bar graphs represent mean values ±SD, n=10 (random fields). The apoptotic rates following exposure to Ver-155008 plus DOX are not statistically significantly different from one to another. **B**, Western blot analysis of human iPS-CMs transduced with ad-GFP, ad-ILK^*WT*^ and ad-ILK^*R211A*^ and then treated for 24 hours with 40 µM of Ver-155008 versus untreated cells in the presence of 1µM DOX. The blot was probed for ILK, Hsp70, Hsc70, Hsp90, pGsk-3β, total Gsk-3β, pAkt, total Akt, total PLN, and GAPDH was used as a loading control.

ILK knockdown using siRNA increased the rate of DOX-induced apoptosis (Figure 5B), whereas there was a concomitant DOX-induced increase in Hsp70 expression (Figure 5C). This result indicates that increased ILK expression is required for cytoprotection against DOX cardiotoxicity, irrespective of the expression levels of Hsp70. The addition of DOX plus a sub-apoptotic dose of Ver-155008 (40 μM) resulted in a high (>60%) apoptosis rate (Figure 6A) in the presence or absence of ILK (ILK^*WT*^ and ILK^*R211A*^) transduction. DOX plus Ver-155008 (40 μM) concomitantly resulted in a reduction in the expression levels of ILK, Hsp70, as well as the total and phospho-specific forms of Gsk-3β and Akt (Figure 6B), whereas there was no discernable effect on the levels of Hsp90 expression. This result suggests that the increased coexpression of ILK and Hsp70 is required to protect against DOX-induced apoptosis. The expression level of phospholamban was also unchanged in response to Hsp70 inhibition plus DOX suggesting that Hsc/p70 exhibits preferential affinity towards Ser/Thr kinase clients. This result is in line with a previous study in which Hsp70 overexpression combined with Hsp70-ATPase inhibition caused a dramatic reduction of Akt levels and increased susceptibility to apoptosis in breast cancer cell lines[21].

## Discussion

Together, our results support a model in which protection against myocardial infarction and DOX cardiotoxicity depends upon a functional module minimally comprised by ILK (WT and R211A) and Hsp70. In this model, protection against DOX cardiotoxicity is dependent upon the induction of Hsp70 by exogenous ILK (WT and R211A) that also requires a functional Hsc/p70-mediated chaperone cycle, such that loss of either ILK or Hsp70 expression levels lowers the threshold for stress-induced apoptosis. This result was observed in the ILK^*R211A*^ activation-resistant mutation, pointing to the scaffolding properties of ILK in mediating stress signaling[14]. In line with our results, ILK was also shown to form a tripartite complex comprised by ILK, endothelial nitric oxide synthase and Hsp90 that was protective against atherogenic injury in a scaffolding- but not ILK kinase activity-dependent manner[22]. 

ILK is a mechanoreceptor protein essential for the modulation of load-dependent cardiac contractility[3,23]. Upregulation of endogenous ILK in human heart promotes an adaptive hypertrophic phenotype[2]. ILK adenoviral gene therapy has been shown to improve remodeling in a rat MI model through upregulation of vascular endothelial growth factor-mediated angiogenesis[24], and to protect against adverse remodeling in chronic DOX-induced cardiomyopathy[25]. This study highlights a newly-identified stress-inducible pathway featuring heat-shock protein 70 that accounts, at least in part, for the observed cytoprotective effects resulting from exogenous delivery of ILK mutations, most prominently the ILK^*R211A*^ mutation. The observed pro-apoptotic effects of ILK knockdown despite preservation of Hsp70 expression levels point to the requirement for ILK *per se* in conferring protection against DOX-induced stress signaling. On the other hand, Hsp70-ATPase inhibition in the presence of DOX led to loss of Hsp70 expression, proteolytic degradation of ILK and increased apoptosis, consistent with a model in which Hsp70-stabilized ILK^*R211A*^ comprises a potently cytoprotective module. 

Our data do not exclude potential cardioprotective effects attributable to Hsp70 proteins that are induced by ILK but that function in non-ILK protein complexes, since Hsp70 is broadly cardioprotective and is an essential component of ischemic preconditioning[26-28]. Additionally, our data do not specifically address the function of non-Hsp70 stress-associated proteins that may function either in concert or independently of ILK. We propose that the broadly upregulated heat-shock transcriptional network shown by microarray analysis at baseline in the ILK^*R211A*^ heart serves to precondition it against ischemic and cardiotoxic stress, at least in part as a result of accelerated protein triage of damaged proteins by the ubiquitin proteasome system[29] and through mitigation of endoplasmic reticulum stress[26]. Together, these data suggest a model in which ILK overexpression induces a chaperone response which, in turn, is broadly cardioprotective against diverse stressors.

It is noteworthy that the ILK^*R211A*^ induced a global gene expression pattern featuring significant enrichment of protein folding pathways based on GO categories. We speculate that the ILK R211A mutation is misfolded as a result of the mutation in its PH domain, which must be sufficiently impaired geometrically to prevent normal membrane binding[6,30]. In that regard, Hsc70 binds all misfolded proteins[31] and was found to specifically co-immunprecipitate with the ILK R211A mutation. Although upregulation of endogenous ILK has been variously implicated as oncogenic or tumor-suppressive, the ILK^*R211A*^ mutation, which is resistant to receptor-mediated activation[6] and to angiotensin II-induced cardiac hypertrophy induction[2], is also either phenotypically inert or growth suppressive in a broad range of cancer lines[7,13]. These findings and the lack of Akt activation by ILK^*R211A*^ during post-MI remodeling shown here are predictive of reduced, off-target oncogenic effects potentially associated with ILK pathway stimulation. These properties of ILK^*R211A*^ suggest its potential therapeutic utility in a wide range of heart diseases. Further, the potency and predicted cardioselectivity of ILK^*R211A*^ advances the broader theme of achieving enhanced efficacy/toxicity profiles through engineered mutations in cytoprotective protein kinases.

## Materials and Methods

### Cardiomyocytes Derived from Human induced Pluripotent Stem Cells (Hu iPSs)

Cryopreserved human induced Pluripotent Stem Cells cardiomyocytes (Hu iPS-CMs) (catalogue no. CMC-100-110-001, Cellular Dynamics International) were thawed and cultured on plastic dishes (coated with 0.1% gelatin) in plating medium (catalogue no. CMM-100-110-001). After 2 days the cultured medium was replaced for the maintenance (catalogue no. CMM-100-210-001) medium for 7 more days prior to ILK adenoviral infection as described. Cardiomyocytes were confirmed contract synchronously at greater than 80% confluency. 

### Preparation of rabbit cardiomyocyte primary cultures

Cardiomyocytes were first isolated from New Zealand adult white rabbit (3–3.5 kg) hearts by enzymatic digestion as we have described previously[32]. After isolation, ventricular cardiomyocytes were placed on a circular laminin-coated glass slide (35 mm diameter) (Bioptechs Inc., USA) that was already immersed in culture medium 199 with Earle's salts (Gibco, Canada) and supplemented with 10% FBS, 10 µM cytosine β-d-arabinofuranoside, 100 IU/mL penicillin, 100 mg/mL streptomycin, and 0.08 mg/mL gentamicin in a 60 mm diameter Petri dish. Culture medium was exchanged once for freshly prepared medium after 24 h in culture. Only cell culture plates with more than 75% rod-shaped viable cardiomyocytes were used in these studies.

### Generation of Transgenic Mice and Adenoviral Constructs

The methods for generation of transgenic animals conveying cardiac-specific over-expression of wild type (ILK^*WT*^) and mutant (ILK^*R211A*^; ILK^*S343D*^) versions of human wild type ILK gene have been previously described[2]. The method used for adenoviral infection of human wild type and ILK^*R211A*^ constructs into Hu iPS-CMs and rabbit cardiomyocytes was performed as described[2,33]. Briefly, cells were cultured to 60%–70% confluency prior to adenovirally mediated infection with serotype 5 adenovirus encoding either human wild-type ILK gene (ad-ILK^*WT*^) or a mutant ILK gene (ad-ILK^*R211A*^) in a bicistronic construct containing GFP as a reporter gene[2]. Control cultured cells were infected with adenovirus containing the reporter gene alone (ad-GFP). Cells were infected at 37°C at multiplicity of infection of 1.5 in IMDM media supplemented with 10% FBS for 24 hours and analyzed 3–5 days after infection. The infection efficiency was confirmed by the expression of GFP.

### Inhibitors

To determine the effects of Hsp70 inhibition, cardiomyocytes were transduced with ad-GFP, ad-ILK^*WT*^ and ad-ILK^*R211A*^ and incubated for 48h before exposure to different concentrations of the specific Hsc/p70-ATPase inhibitor Ver-155008 (catalogue number: 3803, Tocris Bioscience). 

### Research Ethics Board Approval

Regarding animal studies, The Animal Care Committee at the Hospital for Sick Children, which operates in accordance with the Terms of Reference following the Canadian Council on Animal Care Guidelines and federal and provincial regulations/legislations, gave approval to this study. 

### Microarray analysis using Affymetrix GeneChip Hybridization

Microarray analysis using Affymetrix GeneChip Hybridization Experimental design, chip hybridizations, and statistical analyses were done in compliance with the Minimum Information About a Microarray Experiment (MIAME) guidelines. Samples were prepared for hybridization according to standard Affymetrix instructions and performed at the Genomic Core Facility at the Hospital for Sick Children. Total RNA was isolated from 12 mouse heart samples utilizing Trizol Reagent (GIBCO/BRL) following the manufacturer's protocol. The quality of tRNA was assessed using the Agilent 2100 Bioanalyzer (version A.02.01S1232, Agilent Technologies). Only RNA samples with the OD ratio of 1.99-2.0 at 260/280 were used for microarray analysis. A total of 12 hybridizations from four groups of mouse genotypes with and without LAD ligation-induced MI (R211A+, R211A-, R211A+MI, and R211A-MI) were performed using the Mouse MOE 430 2.0 array chip (Affymetrix). Analyses of all data derived from arrays were performed using data obtained from GCOS (GeneChip Operating Software). Pivot tables were imported into GeneSpring GX 11 (Agilent Technologies) and Partek Pro2000 platforms (Partek Inc., St. Louis, MO). Genes from the statistically significant (p <0.05) gene list for the indicated comparisons were used to determine GO terms that were enriched. The p-values were then re-calculated to yield more stringent corrected p-values using Benjamini-Yekutieli to account for multiple GO term testing.

### Data Deposition

Raw data from microarray experiments was submitted to the Gene Expression Omnibus database (http://www.ncbi.nlm.nih.gov/geo) and the GEO accession ID is GSE25729.

### Mouse Myocardial Infarction Model

Permanent proximal LAD ligation was performed as described[34], using 1% isofluorane anesthesia and Buprenorphine at a dosage of .05 -.1 mg/kg for postoperative analgesia.

### Mouse Echocardiography

Echocardiograms evaluating baseline systolic and diastolic function were performed in all mice under general anaesthetic using a Vevo 660 UBM (VisualSonics, Toronto, ON, Canada)[35] [36]. Baseline studies revealed no genotype-specific significant differences in heart rate, stroke volume, cardiac output, fractional shortening, left ventricular dimensions, or pulmonary venous flow patterns. A total of 53 mice underwent ligation of left anterior descending (LAD) coronary artery, including ILK^*R211A*^ (n=13), ILK^*S343D*^ (n=12) and respective littermate controls. Three mice died following surgery (1, ILK^*R211A+*^; 1, ILK^*S343D+*^; and 1 ILK^*R211A-*^), which was not statistically different among genotypes (p=0.89). Echocardiograms were then performed at 28 days following the LAD ligation. LV dimensions (systolic and diastolic) and ejection fraction were determined from the standard parasternal long axis M-mode view. Infarct size was measured by tracing the area of akinesis in the parasternal long axis view, and the area of synchronous contraction on the anterior border of the heart calculated, indicative of viable, non-infarcted myocardium. Differences among transgenic groups were evaluated using a paired analysis adjusting for baseline function and compared using ANOVA. Differences between groups were compared using linear contrasts (PROC GLM) in SAS 9.2 (Cary, NC).

### Western Blots

For Western blot analysis, total and phospho-specifc protein expression was measured in lysates derived from human iPSC-derived cardiomyocytes in culture and from transgenic and control mouse ventricular tissue as described previously[2]. Briefly, cells extracts were prepared by lysing the cells for 20 min on ice in RIPA lysis buffer (150 mM NaCl, 1% Nonidet P40, 0.5% deoxycholate, 0.1% SDS, 50 mM Tris, pH 8.0, and 1mM PMSF). Proteins were visualized by chemiluminescence using either SuperSignal West Pico substrate (Pierce) or SuperSignal West Dura Extended Duration Substrate (Pierce). The expression levels of the proteins were assessed using the following antibodies: ILK (catalogue number: 3856, Cell Signaling Technology), GAPDH (catalogue number: G8795, Sigma-Aldrich), Total Akt (catalogue number: 9272, Cell Signaling Technology), Phospho-Akt (Ser473) (catalogue number: 9271, Cell Signaling Technology), Total GSK-3 β (catalogue number: 9315, Cell Signaling Technology), Phospho-GSK-3 β (Ser9) (catalogue number: 9323, Cell Signaling Technology), Hsp70 (catalogue number: sc-32239, Santa Cruz Biotechnology, Inc.), Hsc70 (catalogue # ab69558, Abcam), Hsp90 (catalogue number: MAB3286, R&D Systems). After incubation with the primary antibody, the blots were washed and incubated for 1 h with the appropriate horseradish peroxidase-conjugated secondary antibody (Jackson ImmunoResearch Laboratories). 

### RNA Interference, Transfection, and Transduction

siRNA against ILK (siILK) and negative control siRNA were purchased from Cell Signaling Technologies. Cells were transfected with siRNAs (130 µM final concentration) using the X-tremeGENE siRNA transfection reagent (Roche Diagnostics, Laval, QC, Canada) according to the manufacturer’s instructions. To induce transduction efficiency the cells were transfected again after 24 hours with the same concentration of siRNA. The cells were analysed at 48 h post-transfection.

### 2-Dimension Isoelectric Focusing (IEF)/SDS PAGE

Immunoprecipitated samples were resolved first by a 5% acrylamide IEF gel with 2% carrier ampholytes (pH 9.5/3.5) as described elsewhere[37]. The immunoprecipitation and control lanes of the IEF gel were excised in segments from top to bottom, inserted for electrophoresis in second dimension SDS PAGE and analyzed by immunoblotting. 

### Mass Spectrometry

The mass spectrometric experiments were performed using an on-line liquid chromatography- tandem mass spectrometry setup using an Agilent 1100 Capillary LC system (Palo Alto, CA) fitted to a LTQ ion trap mass spectrometer (Thermo Electron, San Jose, CA). A C18 pre-column (100µm i.d. x 5.0 cm length) and a µLC analytical column (75 µm x 10 cm) that also served as a µESI emitter were used for the separation of the digested proteins. The mass spectrometer was operated in data-dependent mode automatically cycling through acquisition of a full-scan mass spectrum and three MS/MS spectra recorded sequentially on the three most abundant ions present in the initial MS scan. A dynamic exclusion list time of 1.5 minutes was used. For the reverse phase chromatography, an 80 minute gradient elution from water to acetonitrile, each containing 0.1% FA and 0.02% TFA, was performed at a flow of 200nL/min. All MS/MS spectra were searched against the NCBInr protein database using MASCOT Server (v2.2) (Matrix Science). The search results were analyzed using Scaffold software (Proteome software).

### TUNEL assay

The presence of apoptosis-related DNA strand breaks in Hu iPS-CMs was evaluated by TUNEL assay using the In situ Cell Death Detection kit, POD (Roche Molecular Biochemicals, Mannheim, Germany) according to the manufacturer’s instructions. In short, cells on coverslips were maintained in culture and then fixed with 4% paraformaldehyde for 20 min at room temperature. Following rinsing with PBS, cells were permeabilized with 0.1% Triton-X 100 (Sigma) for 10 min and then supplemented with 3% H_2_O_2_ to quench endogenous peroxidase activity. Thereafter, cells were incubated with TUNEL reaction mixture (terminal deoxynucleoti-dyl transferase and labeled nucleotide mixture). Next, cells were rinsed in PBS and incubated with Converter-POD (anti-fluorescein antibody conjugated with horse-radish peroxidase). Peroxidase activity was then visualized by precipitation of DAB (DAB Substrate, Roche, Mannheim, Germany). Cells containing fragmented nuclear chromatin exhibited a brown nuclear stain. As negative controls, sections were processed without terminal deoxynucleotidyltransferase (TdT) buffer. Apoptotic cells were examined with a Leica DMR microscope. The number of TUNEL-positive cells was counted in at least 10 random fields per treatment. 

### Statistical Analysis

Statistical comparison of ILK-specific effects relied on a paired t test or analysis of variance (ANOVA) followed by the multiple-comparison Bonferroni *t* test to assess differences among groups. Results are given as means ± SD. In tests with one variable only, the t-test was used. Results were considered significant at *P* < 0.05. 

## Supporting Information

Figure S1 **ILK^*R211A*^ induces a robust heat-shock protein transcriptional response.** Unsupervised network map generated using by genes exhibiting significant (p<0.05) changes in expression levels between myocardium derived from ILK^*R211A*^ and littermate controls. Each target is hyperlinked to an interactive html site representing a separate PubMed citation. A highly coherent heat-shock protein (Hsp) response was observed in which 17/25 genes were annotated as Hsp or Hsp-related genes. The networked genes identified *ILK* but did not identify any third party genes that were not determined as significant by *a* *priori* microarray analysis, indicating a highly coherent functional clustering of ILK-associated Hsp genes. For example, upregulation of Hspa8 (human orthologue Hsc70) and Hsc70-interacting genes St13 and *Stip1* imply activation of an Hsc70 chaperone complex. Network map was constructed from Pub Med-derived interactions using Gene Set Analysis, and microarray analysis was performed using Mouse MOE 430 2.0 array chip (Affymetrix), as described in Materials and Methods. Red, upregulated genes; blue, downregulated.
(TIF)

Figure S2 **ILK^*R211A*^ induces a robust heat-shock protein transcriptional response to MI.** Network map of genes showing significantly higher expression of heat-shock related proteins in ILK^*R211A*^ transgenic mouse hearts as compared to those in littermate controls measured 28 days following induction of myocardial infarction. Among 27 of those genes 10 genes were found connected by previous PubMed citations shown hyperlinked to an interactive html site. Analysis method was the same as in Figure S1.
(TIF)

Table S1 **Echocardiographic assessment of cardiac function in ILK^*R211A*^ and ILK^*S343D*^ transgenic mice.** Echocardiographic assessment of cardiac function (mean ± SEM) was performed in transgenic mice conveying ILK activation-resistant (ILK^*R211A*^) and activated (ILK^*S343D*^) mutations and littermate controls at 28 days post-LAD ligation. P values shown were calculated using ANOVA. Pos, genotype-positive; Neg, genotype-negative, littermate controls.
(DOC)

Table S2 **Echocardiographic assessment of infarct size in ILK^*R211A*^ and ILK^*S343D*^ transgenic mice.** Echocardiographic assessment of infarct size was performed (mean ± SEM) in transgenic mice conveying ILK^*R211A*^ and ILK^*S343D*^ mutations and their littermate controls at 28 days post-LAD ligation. Infarct size was taken to be the length of myocardium which was akinetic in the parasternal long axis view. P values shown were calculated using ANOVA. Pos, genotype-positive; Neg, genotype-negative, littermate controls.
(DOC)

Table S3 **Genes upregulated in ILK^*R211A*^ mouse hearts.** List of genes with fold changes and GO annotations was generated using microarray analysis in ILK^*R211A*^ transgenic mouse hearts at baseline according to methods described in Materials and Methods.
(DOC)
