# Supplementary figures and images for: Mutation in Integrin-Linked Kinase (ILKR211A) and Heat-Shock Protein 70 Comprise a Broadly Cardioprotective Complex
Source: PLoS One. 2013 Nov 18;8(11):e77331. doi: 10.1371/journal.pone.0077331 (PMC3832499; doi:10.1371/journal.pone.0077331)

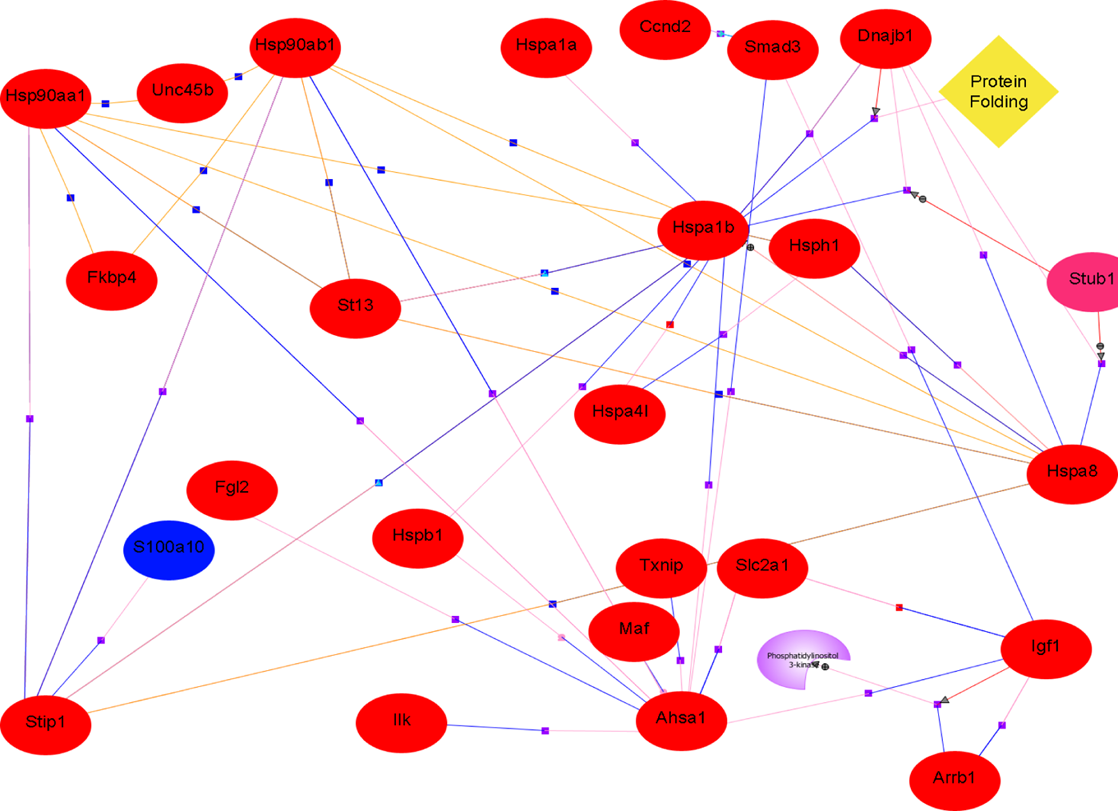

Supplement: Figure S1 — ILKR211A induces a robust heat-shock protein transcriptional response. Unsupervised network map generated using by genes exhibiting significant (p<0.05) changes in expression levels between myocardium derived from ILKR211A and littermate controls. Each target is hyperlinked to an interactive html site representing a separate PubMed citation. A highly coherent heat-shock protein (Hsp) response was observed in which 17/25 genes were annotated as Hsp or Hsp-related genes. The networked genes identified ILK but did not identify any third party genes that were not determined as significant by a priori microarray analysis, indicating a highly coherent functional clustering of ILK-associated Hsp genes. For example, upregulation of Hspa8 (human orthologue Hsc70) and Hsc70-interacting genes St13 and Stip1 imply activation of an Hsc70 chaperone complex. Network map was constructed from Pub Med-derived interactions using Gene Set Analysis, and microarray analysis was performed using Mouse MOE 430 2.0 array chip (Affymetrix), as described in Materials and Methods. Red, upregulated genes; blue, downregulated. (TIF) [file pone.0077331.s001.tif]

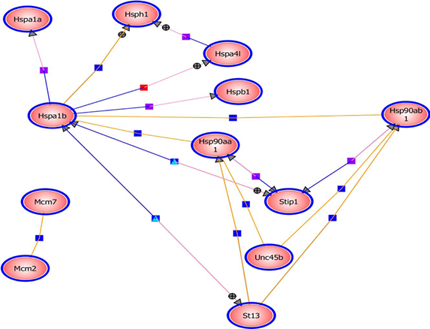

Supplement: Figure S2 — ILKR211A induces a robust heat-shock protein transcriptional response to MI. Network map of genes showing significantly higher expression of heat-shock related proteins in ILKR211A transgenic mouse hearts as compared to those in littermate controls measured 28 days following induction of myocardial infarction. Among 27 of those genes 10 genes were found connected by previous PubMed citations shown hyperlinked to an interactive html site. Analysis method was the same as in Figure S1. (TIF) [file pone.0077331.s002.tif]
