# Supplementary material for: Mutation in Integrin-Linked Kinase (ILKR211A) and Heat-Shock Protein 70 Comprise a Broadly Cardioprotective Complex
Source: PLoS One. 2013 Nov 18;8(11):e77331. doi: 10.1371/journal.pone.0077331 (PMC3832499; doi:10.1371/journal.pone.0077331)
Supplement: Table S3 — Genes upregulated in ILKR211A mouse hearts. List of genes with fold changes and GO annotations was generated using microarray analysis in ILKR211A transgenic mouse hearts at baseline according to methods described in Materials and Methods. (DOC) [file pone.0077331.s005.doc]

| **Gene Symbol** | **Gene Name** | **Fold change** | **Gene Ontology Biological Process** |
| --- | --- | --- | --- |
| **Ilk** | Integrin-linked kinase | 13.3 | ureteric bud branching // protein amino acid phosphorylation // integrin-mediated signaling pathway // positive regulation of cell proliferation // regulation of signal transduction //establishment or maintenance of epithelial cell apical/basal polarity |
| **Ifi202b** | Interferon-activated gene 202b | 4.1 | protein binding |
| **Hspa1b** | Hsp70 | 3.4 | telomere maintenance // DNA repair // anti-apoptosis // response to stress // response to heat // negative regulation of caspase activity |
| **Hspa1a** | Hsp70-3 | 3.1 | telomere maintenance // DNA repair // response to stress // response to heat |
| **Hsph1** | Hsp105 | 2.7 | response to stress //chaperone mediated protein folding requiring cofactor |
| **Uap1** | UDP-N-acetylglucosamine pyrophosphorylase 1 | 2.1 | metabolic process |
| **Atp1a2** | ATPase, Na+/K+ transporting, alpha 2 polypeptide | 2.1 | neurotransmitter uptake // regulation of the force of heart contraction // regulation of respiratory gaseous exchange by neurological process // ATP biosynthetic process // ion transport // reduction of cytosolic calcium ion concentration |
| **Hsp90aa1** | Hsp90 | 2.0 | protein folding // nitric oxide biosynthetic process // response to stress // response to unfolded protein // protein refolding // positive regulation of cytotoxic T cell differentiation |
| **LOC677213** | similar to U2AF homology motif (UHM) kinase 1 | 1.9 | protein amino acid phosphorylation // cell cycle arrest // peptidyl-serine phosphorylation // protein amino acid autophosphorylation // regulation of protein export from nucleus |
| **Myh7** | myosin, heavy polypeptide 7, cardiac muscle, beta | 1.8 | response to reactive oxygen species // ATP catabolic process // striated muscle contraction |
| **Abhd3** | abhydrolase domain containing 3 | 1.7 | carboxylesterase activity // hydrolase activity |
| **Dnaja1** | Hsp40 homolog, subfamily A, member 1 | 1.6 | protein folding // spermatogenesis // sperm motility // androgen receptor signaling pathway |
| **Hspb1** | Hsp25 | 1.6 | response to stress // response to heat |
| **Hspa8** | Hsp70 cognate protein | 1.6 | protein folding // response to stress // chaperone mediated protein folding requiring cofactor // regulation of cell cycle |
| **Obfc2a** | oligonucleotide/oligosaccharide-binding fold containing 2A | 1.6 | nucleic acid binding // single-stranded DNA binding // RNA binding |
| **P4ha1** | proline 4-hydroxylase, alpha 1 polypeptide | 1.5 | peptidyl-proline hydroxylation to 4-hydroxy-L-proline // protein metabolic process // collagen fibril organization // oxidation reduction |
| **Thrsp** | thyroid hormone-responsive protein | 1.5 | protein binding |
| **Clu** | complement lysis inhibitor | 1.5 | anti-apoptosis // response to oxidative stress // cell death // positive regulation of cell proliferation // endocrine pancreas development // positive regulation of cell differentiation // neuron projection morphogenesis |
| **Txnip** | thioredoxin binding protein-2 | 1.5 | 0006350 // transcription // inferred from electronic annotation /// 0006355 // regulation of transcription, DNA-dependent // inferred from electronic annotation /// 0006979 // response to oxidative stress // inferred from direct assay /// 0007049 // cell cycle // inferred from electronic annotation /// 0030216 // keratinocyte differentiation // inferred from electronic annotation /// 0048008 // platelet-derived growth factor receptor signaling pathway // inferred from mutant phenotype |
| **Hspa4l** | Hsp70 protein 4-like | 1.5 | protein folding // response to stress // response to unfolded protein |
| **Ahsa2** | AHA1, activator of heat shock protein ATPase homolog 2 | 1.5 | response to stress |
| **Hspe1** | heat shock protein 1 (chaperonin 10) | 1.5 | protein folding // response to stress |
| **Dnajb1** | Hsp40 homolog, subfamily B, member 1 | 1.5 | protein folding // response to stress // chaperone mediated protein folding requiring cofactor |
