# Supplementary material for: Mutation in Integrin-Linked Kinase (ILKR211A) and Heat-Shock Protein 70 Comprise a Broadly Cardioprotective Complex
Source: PLoS One. 2013 Nov 18;8(11):e77331. doi: 10.1371/journal.pone.0077331 (PMC3832499; doi:10.1371/journal.pone.0077331)
Supplement: Supplementary file 3 [file pone.0077331.s003.doc]

ECHO Variable	**ILK**^{R211A}			**ILK**^{S434D}		
	**Pos (n=13)**	**Neg (n=13)**	**P-Value**	**Pos (n=12)**	**Neg (n=12)**	**P-Value**
Stroke Volume	27.3 ± 1.3	23 ± 1.7	0.04	19.7 ± 1.3	20.2 ± 1.6	0.53
Heart Rate	394 ± 12.5	441 ± 28	0.02	469 ± 8.3	454 ± 11.7	0.43
Cardiac Output	8.9 ± 85	9.2 ± 1.05	0.84	9.2 ± 0.63	10.2 ± 0.81	0.51
End-Diastolic Dimension	4.9 ± 0.19	5.2 ± 0.29	0.47	5.3 ± 0.23	5.0 ± 0.18	0.46
End-Systolic Dimension	4.1 ± 0.24	4.5 ± 0.37	0.53	4.8 ± 0.28	4.2 ± 0.22	0.33
Fractional Shortening	16.2 ± 2.2	15.6 ± 2.2	0.91	10.2 ± 1.85	15.5 ± 1.65	0.3
