# Supplementary material for: Mutation in Integrin-Linked Kinase (ILKR211A) and Heat-Shock Protein 70 Comprise a Broadly Cardioprotective Complex
Source: PLoS One. 2013 Nov 18;8(11):e77331. doi: 10.1371/journal.pone.0077331 (PMC3832499; doi:10.1371/journal.pone.0077331)
Supplement: Supplementary file 4 [file pone.0077331.s004.doc]

ECHO Variable	**ILK^{R211A}**			**ILK^{S434D}**		
	**Pos (n=13)**	**Neg (n=13)**	**P-Value**	**Pos (n=12)**	**Neg (n=12)**	**P-Value**
Infarct dimension	5.4 ± 0.22	7.2 ± 0.34	0.04	6.5 ± 0.31	7.6 ± 0.27	0.12
Viable myocardium	5.7 ± 0.42	4.25 ± 0.3	0.001	5.1 ± 0.25	4.2 ± 0.21	0.05
